# Is Alcohol Consumption Associated with a Lower Risk of Cardiovascular Events in Patients Treated with Statins? An Observational Real-World Experience

**DOI:** 10.3390/jcm11164797

**Published:** 2022-08-17

**Authors:** Jeffrey L. Anderson, Viet T. Le, Tami L. Bair, Joseph B. Muhlestein, Kirk U. Knowlton, Benjamin D. Horne

**Affiliations:** 1 The Intermountain Medical Center Heart Institute, Salt Lake City, UT 84107, USA; 2 The Cardiology Division, Department of Internal Medicine, University of Utah School of Medicine, Salt Lake City, UT 84108, USA; 3 Rocky Mountain University of Health Professions, Provo, UT 84606, USA; 4 Division of Cardiovascular Medicine, Department of Medicine, Stanford University, Stanford, CA 94305, USA

**Keywords:** statins, alcohol, alcohol consumption, cardiovascular risk

## Abstract

Alcohol consumption has long been associated with cardiovascular (CV) benefit, but it also has adverse potential. Statins are currently widely used for CV prevention. We evaluated whether alcohol use is associated with lower CV risk in patients on statins. We searched Intermountain Medical Center cardiac catheterization laboratory medical records for patients with a prescription history of statin use or non-use and a self-report of alcohol use or non-use. Alcohol and statin prescription data were available together with long-term (mean [SD], 4.4 [2.4] years) major adverse CV events (MACE, including death, myocardial infarction, stroke, and heart failure hospitalizations) in 1701 patients at primary and 3266 patients at secondary CV risk. MACE rates were lower for primary prevention alcohol users than non-users not on statins (adjusted hazard ratio [adj-HR] 0.50 (95% CI 0.33, 0.78, *p* = 0.002), but not for those on statins (adj-HR 0.84, CI 0.54, 1.32, *p* = 0.45). MACE rates for secondary prevention were not reduced by alcohol consumption either in statin non-users or users (adj HR 1.18, CI 0.85, 1.64, *p* = 0.33; adj HR 1.08, CI 0.87, 1.35, *p* = 0.45, respectively). These findings, together with other recent supportive studies, can help inform personal choices in alcohol consumption and professional society recommendations for CV prevention.

## 1. Introduction

Cardiovascular (CV) disease remains the leading global cause of mortality [1,2]. Consequently, CV disease represents a key target for preventive measures through lifestyle and medical interventions [3,4]. Alcohol consumption was long associated with cardiovascular preventive benefits in observational epidemiological studies [5,6], but it also has well-known adverse potential, and no randomized controlled trials assessed its benefit/risk ratio [7].

As preventive and treatment therapies evolve and advance, older therapies often showed diminished benefit, and indications for their use are more limited. In CV medicine, examples of this phenomenon include a diminished or more restricted role for aspirin for primary prevention [8], beta-blocker therapy for patients with stable coronary artery disease (CAD) [9], and digitalis glycosides for treatment of heart failure (HF) [10]. In the current prevention era, statins are widely used as safe and effective CV preventive therapies [11,12,13]. We asked the question whether alcohol use would still be associated with a lower risk of adverse CV events in patients taking statins for primary or secondary prevention.

## 2. Materials and Methods

**Study aim:** The primary aim of the study was to assess whether alcohol consumption is associated with CV benefit in patients prescribed or not prescribed statins for primary CV prevention in Intermountain Medical Center. The secondary aim was to evaluate this question in patients at secondary CV risk.

**Study design and IRB approval:** The study hypothesis was prespecified prior to study analyses and was applied to a prospectively collected observational registry (the INSPIRE registry: clinicaltrials.gov NCT02450006) using patient-reported alcohol consumption data that were merged with electronic medical records of Intermountain Healthcare. Subjects consented to participation in the registry to provide survey information, biobanked plasma and DNA samples, and linked medical records, and this study was approved by the Intermountain Healthcare Institutional Review Board.

**Study population and data source:** Intermountain Healthcare is a nonprofit, integrated healthcare system that, at the time of this study, included 24 hospitals and 215 clinics in Utah, Idaho, and Nevada. Intermountain Healthcare has a long-standing integrated electronic medical records system and a complementary, detailed catheterization laboratory records database. Intermountain Medical Center is the flagship tertiary referral hospital of Intermountain Healthcare.

Patients undergoing cardiac catheterization and coronary angiography at Intermountain Medical Center were approached to enroll in the Intermountain INSPIRE Registry, which involves gaining written permission to obtain a blood sample for DNA and plasma banking-related medical research, and for linking to clinical outcomes through the Intermountain Healthcare electronic data warehouse (EDW). Patients undergoing angiography were also invited to complete a customized questionnaire focusing on key measures of lifestyle potentially related to CV health and outcomes [14]. Specific to the question of alcohol consumption, the questionnaire asked, “In the past year, how many drinks of alcoholic beverages have you had per week?” The possible responses were the following: None, <1 drink per week,1–7 drinks per week, >7 drinks per week, or decline to answer. “None” was defined as non-use and any of the <1 drink, 1–7 drinks, or >7 drinks per week responses was considered to represent alcohol consumption. Those who declined to answer were excluded from the analysis.

Those providing written consent and completing and returning the demographic- and lifestyle-related questionnaire formed the current study population. The dates of enrollment were 2013 to 2020.

The study population was then stratified into those at primary risk (no clinical history of myocardial infarction [MI], coronary revascularization procedure, or significant coronary artery stenosis at current or prior angiography [defined as ≥70% coronary artery stenosis]), and those at secondary risk (i.e., having had at least one of these criteria).

**Study outcomes and hypothesis testing:** Major adverse cardiovascular events (MACE) were defined as the occurrence after study enrollment (defined as the date of questionnaire completion) of an EDW documented MI, ischemic stroke, heart failure (HF) hospitalization, or all-cause mortality. (Our records did not consistently contain a specific cause of death.) Hypothesis testing included a comparison of patients on statins with those off statins who were alcohol users or non-users in the primary prevention cohort (primary comparisons) and the secondary prevention cohort (secondary comparisons). MACE was determined and compared by alcohol and statin use.

We prospectively hypothesized that alcohol would not reduce the MACE risk in statin users. We also tested whether an alcohol-related benefit would be observed in non-statin users, especially in the primary prevention cohort.

**Statistics:** Chi-square tests and *t*-tests were used to examine differences in baseline characteristics and medications for patients on/off statins and on/off alcohol. Cox proportional hazards regression was used to examine outcomes, adjusted for baseline differences, comparing on/off alcohol use and on/off statin use for primary and secondary prevention. Hazard ratios were adjusted for age, sex, race, ethnicity, marital status, education, income, employment status, general physical activity, walking, cycling, body mass index, smoking status, medical history (i.e., hypertension, hyperlipidemia, diabetes, atrial fibrillation, MI, HF, stroke, cancer, COPD, renal failure, peripheral vascular disease, dementia, and depression), family history of early CAD, hospital presentation (stable, unstable angina, or acute MI), and baseline medications (including aspirin, statins, beta-blockers, angiotensin-converting enzyme inhibitors, angiotensin receptor blockers, antiplatelets, anticoagulants, calcium channel blockers, anti-diabetic medications, digoxin, and diuretics). Patients with CAD also had associations adjusted for prior CAD diagnosis, number of diseased coronaries (left main involvement was considered 2 vessel disease), and treatment type (medical only, percutaneous coronary intervention, or coronary bypass surgery).

## 3. Results

**Characteristics of the study population:** Alcohol and statin prescription data were available together with long-term (mean [SD], 4.4 [2.4] years) MACE outcomes in 1701 patients at primary CV risk (4.0 [2.3] years of follow-up) and 3266 patients at secondary CV risk (4.6 [2.4] years of follow-up) (total N = 4967). Age, sex, and frequency distributions by alcohol status and statin prescription are shown in Table 1 for patients at primary CV risk and in Table 2 for patients at secondary CV risk. In the primary prevention group (average age, 57.9 years; 52.4% male), 28% of study patients were prescribed a statin. In the secondary prevention group (average age, 66.2 years; 67.8% male), 64.5% had a documented statin prescription.

Major coronary risk factors (hyperlipidemia, hypertension, diabetes, smoking) were present in 19.8%, 25.9%, 21.8%, and 2.3% at primary risk, respectively, and 54.4%, 56.8%, 45.0%, and 8.6% at secondary risk (Table 3). At coronary angiography, no/negligible, mild, and obstructive (≥70% stenosis) CAD was found in n = 1701 (100%), 0 (0%), and 0 (0%) of the study population at primary risk, and n = 963 (29.5%), 438 (13.4%), and 1865 (57.1%) at secondary risk (Table 3). Past h/o stroke and MI were present in 4.5% and 0%, respectively, of the primary risk population and 7.1% and 12.4% of the secondary risk population (Table 3).

**Figure 1:**
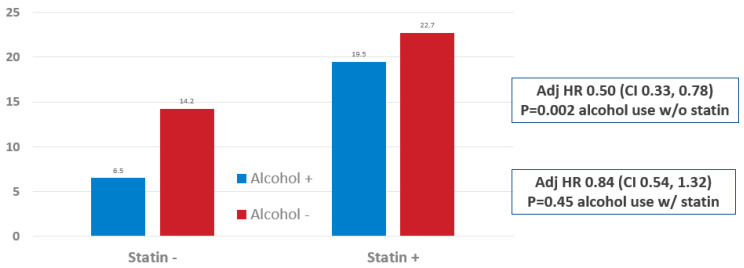
MACE Outcomes in Primary Prevention Patients by Alcohol and Statin Status. Alcohol+ = alcohol user. Alcohol− = Alcohol non-user.

**Long-term outcomes by statin and alcohol status in the primary prevention cohort:** MACE rates during follow-up were 6.5% and 14.2% for primary prevention alcohol users and non-users, respectively (Figure 1), with an adjusted hazard ratio (adj-HR) of 0.50 (95% CI 0.33, 0.78), *p* = 0.002, for those not on statins. In contrast, rates were 19.5% and 22.7%, adj-HR 0.84 (CI 0.54, 1.32), *p* = 0.45, for those on statins (Figure 1). Individual components of MACE are shown in Table 3.

**Figure 2:**
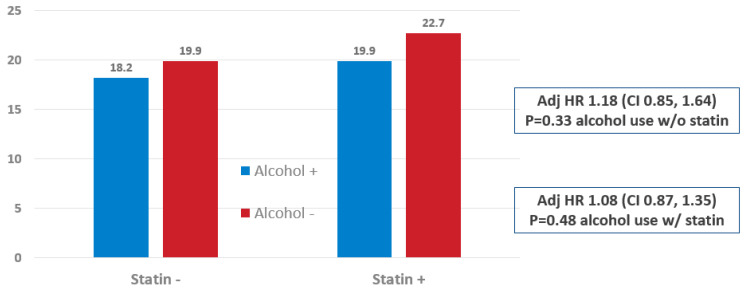
MACE Outcomes in Secondary Prevention Patients by Alcohol and Statin Status. Alcohol+ = alcohol user. Alcohol− = Alcohol non-user.

**Long-term outcomes by statin and alcohol status in the secondary prevention cohort:** For secondary prevention, MACE rates for alcohol users and non-users were, respectively, 18.2% and 19.9% for patients not on a statin, adj HR 1.18 (CI 0.85, 1.64), *p* = 0.33, and 19.9% and 22.7%, adj HR 1.08 (CI 0.87, 1.35), *p* = 0.45, for those on statins (Figure 2). Individual components of MACE are shown in Table 3.

**Tertiary and exploratory analyses:** An analysis also was performed by dose of alcohol (0 drinks/day, <2 drinks/day, or ≥2 drinks/day) in the primary prevention population where n = 56 patients not taking statins reported ≥2 drinks/day and n = 22 taking statins had ≥2 drinks/day. In patients on no statins, those consuming alcohol but <2 drinks/day had lower MACE risk compared with non-drinkers, with 5.8% vs. 14.2% MACE (adj HR 0.47 [CI 0.29, 0.75], *p* = 0.002), while ≥2 drinks/day was not different to none with 10.7% vs. 14.2% MACE (adj HR 0.82 [CI 0.36, 1.90], *p* = 0.65). For patients taking statins, MACE was 22.7%, 21.1%, and 9.1%, respectively, for no alcohol, <2 drinks/day (adj HR 0.998 vs. non-drinkers [CI 0.65, 1.54], *p* = 0.99). Too few in this subgroup consumed ≥2 drinks/day for valid statistical comparison.

## 4. Discussion

**Summary of Study Findings:** In a prespecified analysis of this prospectively collected database, we asked the question whether alcohol in statin-treated patients further lowers cardiovascular risk either for primary or secondary CV prevention. The results of our analysis show no significant incremental benefit in either risk cohort. We did find evidence of a benefit of alcohol consumption in the primary prevention cohort not taking a statin. This latter result could be viewed as a ‘positive control’, consistent with long-standing epidemiological evidence of benefit dating to the pre-statin era. However, we did not find a benefit of alcohol in untreated patients in the secondary risk population, perhaps because of other aggressive preventive measures.

**Literature Insights:** Epidemiological studies dating back several decades have repeatedly reported light to moderate consumption of alcohol to be associated with a lower risk of CV disease compared with abstinence or heavy intake, which has suggested a ‘U-shaped’ dose–response curve [5,6,15,16,17,18]. However, controversy arises because of reports that light–moderate alcohol intake is also associated with favorable lifestyle, behavioral, and socioeconomic factors, which could explain the apparent beneficial alcohol-related association [19]. Mendelian randomization methodology, based on genetic variants predictive of alcohol consumption, represents a promising approach for the testing of causality apart from environmental factors [20,21,22]. Recently, Biddinger et al., studied 371,463 participants enrolled in the UK Biobank to test for confounding associations between alcohol intake and CV diseases and risk factors, including by nonlinear mendelian randomization [23]. They found that coincident favorable lifestyle factors attenuated or nullified the benefits of modest alcohol intake and suggested that any and all amounts of alcohol consumption were associated with increased CV risk, with risk exponentially increasing from minor (with modest intake) to several fold greater, with intake of 21 or more drinks per week. Nonlinear risk increases were noted not only for CAD but also for blood pressure, lipids, and atrial fibrillation. Consistent with their conclusion of lack of benefit with even modest amounts of alcohol, the World Heart Federation recently advocated against any consumption of alcohol for health benefits [24], citing the extensive 2018 *Lancet* report of ‘Alcohol use and burden for 195 countries and territories’ [25].

These recent reports both support our findings and go beyond, raising the possibility that the apparent benefit of alcohol in our primary prevention, non-statin cohort might be explained by uncorrected confounding of other associated beneficial factors.

**Mechanistic Considerations:** Several potential mechanisms were proposed for the reported CV preventive benefits of alcoholic beverages, including antioxidant (e.g., in red wine), anticoagulant, and psychosocial factors (stress reducing). In the current era of CV prevention, statins represent a highly beneficial, cost-effective preventive strategy.

Medical history is replete with examples of therapeutics that, over time, offer only attenuated benefits and, consequently, more restricted indications. Digitalis, beta-blockers, and aspirin (for primary prevention) represent just a few examples. With controversy surrounding the role of alcohol in CV risk reduction, we asked the question whether alcohol has a benefit incremental to statins in primary and/or secondary prevention of cardiovascular (CV) disease, and we found a negative answer.

**Clinical Implications:** Given controversies associated with the modest evidence for the benefits of alcohol and the known harmful potential of excessive alcohol intake, how to advise patients with questions about alcohol consumption is challenging and does not have a current consensus answer. The 2021 ESC Guidelines on Cardiovascular Disease Prevention in Clinical Practice advise limiting alcohol intake to less than 100 g per week, while not advocating it as a preventive measure (4). The 2019 ACC/AHA Guideline on the Primary Prevention of Cardiovascular Disease recommends limiting alcohol intake to ≤2 drinks/day in men and ≤1 drink/day in women with hypertension, again while neither advocating nor proscribing it for coronary heart prevention (3). US general dietary guidelines recommend similar upper dose thresholds, albeit with limited evidence for overall safety of those thresholds [26].

Our study findings, indicating no benefit in statin users, and the UK Biobank-related [23] and World Health Federation publications [24], suggest that an even stronger message may be appropriate to patients against the use of alcohol specifically for CV prevention. While modest alcohol consumption appears to adversely impact CV risk only modestly, alcohol does not appear to provide incremental benefit over associated healthy lifestyle habits and evidence-based medical therapies, such as statins.

**Strengths and Limitations:** The study has the strength of using a prospectively acquired database focused on lifestyle measures relevant to CV prevention and a moderately long follow-up period. Another strength is angiographic determination of baseline coronary status, allowing for precise assignment of primary versus secondary prevention cohorts. However, it does have the limitations of all observational studies, including the possibility of residual confounding influencing study results despite adjusted analyses. Another potential limitation is dependence on the accuracy of patient assessment of alcohol consumption. However, it is likely that any discrepancies would tend to be toward underestimates rather than overestimates of intake and tend to nudge results toward the null rather than inflating them. The study is of moderate size, which is adequate for the primary analyses, but it lacks high power for some secondary and tertiary analyses (e.g., alcohol dose–response relationships). Moreover, it cannot exclude small advantages (e.g., of the order of 10%) in MACE reduction by alcohol in the setting of statin use. Its restriction to a single healthcare system increases internal consistency, but it may limit applicability to other systems with differing racial/ethnic make-up and prevention approaches.

## 5. Conclusions

In this single-center healthcare population, alcohol consumption was associated with lower risk of MACE in statin untreated primary prevention patients, but it showed no incremental benefit when added to statins for primary prevention and no benefit with or without statin use for secondary prevention. In keeping with other recent and supportive reports, these findings can help to inform personal choices in alcohol consumption and professional society recommendations for cardiovascular prevention.

## Figures and Tables

**Table 1:** Frequencies by Alcohol and Statin Category, together with Age and Sex in Patients at Primary Cardiovascular Risk.

	Statin—Yes	Statin—No
Alcohol—Yes		
N patients	164	416
Age, mean y (SD)	62 (11)	54 (16)
Sex, % male	67.7%	57.2%
Alcohol—No		
N patients	313	808
Age, mean y (SD)	64 (13)	57 (17)
Sex, % male	53.7%	46.4%

**Table 2:** Frequencies by Alcohol and Statin Category, together with Age and Sex in Patients at Secondary Cardiovascular Risk.

	Statin—Yes	Statin—No
Alcohol—Yes		
N patients	599	292
Age, mean y (SD)	64 (12)	63 (14)
Sex, % male	75.9%	67.8%
Alcohol—No		
N patients	1509	866
Age, mean y (SD)	67 (11)	67 (13)
Sex, % male	69.6%	58.9%

**Table 3:** Major coronary risk factors and baseline history of MACE by primary or secondary prevention status.

Characteristic or	Primary Prevention			Secondary Prevention		
Outcome	Overall	Alcohol+	Alcohol−	Overall	Alcohol+	Alcohol−
*Baseline Characteristics*						
Hyperlipidemia	19.8%	16.7%	21.3% *	54.4%	55.0%	54.2%
Hypertension	25.9%	23.4%	27.2%	56.8%	54.7%	57.5%
Diabetes	21.8%	14.1%	25.8% †	45.0%	35.6%	48.5% †
Smoking	2.3%	3.8%	1.5% *	8.6%	14.4%	6.4% †
Family History	4.5%	5.2%	4.1%	19.4%	19.1%	19.5%
Prior CAD	0%	0%	0%	76.2%	70.3%	78.4% †
Prior MI	0%	0%	0%	12.4%	10.0%	13.3% *
Prior Stroke	4.5%	4.3%	4.6%	7.1%	4.7%	8.0% †
HF History	9.8%	9.5%	9.9%	13.8%	11.0%	14.9% *
Atrial Fibrillation	43.7%	44.8%	43.2%	40.8%	35.7%	42.7% †
Renal Failure	0.5%	0.0%	0.7% *	2.2%	2.1%	2.3%
PVD	19.9%	16.9%	21.5% *	39.2%	34.0%	41.1% †
Angiography: Obstructive CAD						
None	100%	100%	100%	29.5%	30.0%	29.3%
Mild	0%	0%	0%	13.4%	14.4%	13.1%
Significant	0%	0%	0%	57.1%	55.6%	57.6%
Hospital Treatment Modality						
Medical Only	100%	100%	100%	71.4%	70.3%	71.9%
PCI	0%	0%	0%	25.3%	26.1%	25.0%
CABG	0%	0%	0%	3.2%	3.5%	3.1%
*Longitudinal MACE Outcomes*						
MI	0.9%	0.5%	1.0%	6.2%	5.7%	6.4%
Stroke	5.4%	4.0%	6.1%	6.2%	6.3%	6.1%
HF Hospitalization	3.4%	2.3%	4.0%	5.7%	4.8%	6.1%
All-Cause Mortality	5.3%	3.4%	6.3% *	9.7%	8.2%	10.2%

Alcohol+ = alcholol user. Alcohol− = Alcohol non-user. * *p* ≤ 0.05 (and *p* ≥ 0.001) for alcohol− vs. alcohol+; † *p* < 0.001 for alcohol− vs. alcohol+.

## Data Availability

The data underlying this article cannot be shared publicly due to US privacy laws. Data are available upon reasonable request to the corresponding author.
